# Clinical significance of CXCL17 in cervical cancer

**DOI:** 10.1097/MD.0000000000046178

**Published:** 2026-01-02

**Authors:** Jingming Zhai, Tianshu Pang, Yanhui An, Jianguang Wang, Xiaokang Zhang, Longhui Zhang, Like Zhang, Lujia Dong

**Affiliations:** a Department of General Surgery, The First Affiliated Hospital and College of Clinical Medicine, Henan University of Science and Technology, Luoyang, China; b Health Management Center, The First Affiliated Hospital of Henan University of Science and Technology, Luoyang, China.

**Keywords:** cervical cancer, CXCL17, E-cadherin, EMT, snail, vimentin

## Abstract

As an acknowledged inflammatory cytokine, CXCL17 is also participated in various malignancies, including pancreatic ductal adenocarcinoma, lung cancer, colon cancer, and thyroid carcinoma. Nevertheless, it is still unclear whether CXCL17 is correlated with tumorigenesis and advancement of cervical cancer. The aim of this study was to study the clinical significance of CXCL17 in cervical cancer patients and its associations with epithelial-to-mesenchymal transition (EMT) markers. The present study analyzed the CXCL17 expression pattern in cervical cancer and investigated its correlation with clinicopathological parameters in 80 cervical cancer cases. Furthermore, the correlation between CXCL17 and EMT markers were evaluated. The results showed CXCL17 expression was positive in 67.50% (54/80) cases. Our results revealed CXCL17 expression was significantly correlated with lymph node metastasis and clinical stage (*P* < .05). GPR35 expression was significantly enhanced in cervical cancer tissues. More importantly, CXCL17 expression was correlated with snail or vimentin expression. Simultaneously, CXCL17 expression had a negative correlation with E-cadherin expression. These results demonstrated the close correlation between CXCL17 expression and EMT markers. Our findings revealed that CXCL17 promotes advancement of cervical cancer via influence of some critical EMT markers.

## 1. Introduction

The management of cervical cancer has advanced significantly over recent decades, driven by improvements in early diagnosis, surgical techniques, and adjuvant therapies.^[1]^ Many trials with promising results are currently ongoing to establish the role of antibody-drug conjugates and new combination strategies for the management of metastatic and recurrent cervical cancer.^[2]^ However, in developing countries, new cases of cervical cancer were 452,000 and ranked the second most common form of malignancy among women.^[3]^ An estimated 100,700 new cases and 26,400 deaths from cervical cancer occurred in China in 2014.^[4]^ Taking into account the great difficulty for detecting this disease in early stage, the high mortality rates of cervical cancer is still high, especially in rural areas. Therefore, it is of great significance for observing novel molecular targets underlying progression of cervical cancer.

CXCL17 is a member of the CXC chemokine family that attracts myeloid cells. It is a novel chemokine made up of 119 amino acids.^[5,6]^ CXCL17 promotes angiogenesis, metastasis, and cell proliferation in malignancies.^[7–12]^ For patients with endometrial cancer, the overall survival (OS) of patients with high CXCL17 expression was significantly higher than that low CXCL17 expression.^[13]^ Interestingly, Wang et al reported that CXCL17 promotes cell metastasis and inhibits autophagy via the LKB1-AMPK pathway in hepatocellular carcinoma.^[14]^ These studies had elucidated the roles of CXCL17 in various cancers, the effect of CXCL17 on cervical cancer is not clearly understood.

In the present study, on the basis of a discovery cohort of 80 cervical cancer specimens, we explored the expression pattern of CXCL17 and GPR35 in cervical cancer cases. Down-regulation of E-cadherin expression is a hallmark event of epithelial-to-mesenchymal transition (EMT), accompanied by high expression of N-cadherin and vimentin proteins. By studying the correlations between CXCL17 and some EMT markers, we also investigated the role of CXCL17 in EMT of cervical cancer.

## 2. Materials and methods

### 2.1. Patients and tissue samples

Eighty paraffin-embedded cervical cancer specimens (HPV-associated squamous cell carcinoma) and 25 noncancerous cervical epithelial tissues were obtained from the Department of Pathology, The First Affiliated Hospital and College of Clinical Medicine, Henan University of Science and Technology between January 2012 and January 2022. Clinicopathological factors, such as age, tumor size, clinical stage, and lymph node metastasis were listed in Table 1. The informed consent of this study was obtained by each patient. The study was approved by the Medical Ethics Committee of The First Affiliated Hospital and College of Clinical Medicine, Henan University of Science and Technology.

**Table 1:** Correlation between CXCL17 and clinicopathological parameters in cervical cancer patients.

Clinical parameter	N	CXCL17 expression		*P* value
		Positive	Negative	
Age (yr)				
<50	32	22	10	
≥50	48	32	16	.845
Tumor size				
<4 cm	43	31	12	
≥4 cm	37	23	14	.344
Clinical stage				
I + IIa	45	26	19	
IIb + III	35	28	7	.035
Lymph node metastasis				
Absent	42	24	18	
Present	38	30	8	.038

* <.05.

### 2.2. Immunohistochemistry and immunohistochemical assessment

Firstly, paraffin-embedded donor tissue blocks were overlaid for tissue microarrays. Cylindrical tissue (diameter, 0.5 mm) were punctured from specific areas of the donor tissue blocks and re‑embedded into a recipient paraffin block at the designated location.

The blocks were cut into 5-µm sections and deparaffinized by routine procedures. Then, the slides were microwaved in citrate buffer for antigen retrieval for 5 min. Sections were then incubated with primary antibodies overnight at 4°C: CXCL17 (18108-1-AP, 1:200 dilution; Proteintech), GPR35 (55248-1-AP, 1:150 dilution; Proteintech), E-cadherin (20874-1-AP, 1:200 dilution; Proteintech), Vimentin (10366-1-AP, 1:100 dilution; Proteintech), and Snail (13099-1-AP, 1:200 dilution; Proteintech). The sections were incubated with a secondary antibody (Maxim-Bio, Fuzhou, China). After labeling by diaminobenzidine (Maxim-Bio), sections were counterstained with hematoxylin. Sections were scored according to the intensity (0, no staining; 1, weak staining; 2, moderate staining; 3, strong staining) and the percentage (extent staining) of cancer cells that were stained (0, no positive cells; 1, <10% of tumor cells stained; 2, 10% to 50% of tumor cells stained; 3, >50% of tumor cells stained; 4, >75% of cells staining positive). If the product of multiplication between staining intensity and the percentage of positive cells is ≥2, it is regarded as immunoreaction positive (+). Furthermore, in all positive cases, IHC results ≦ 4 and ≦ 6 were defined as low and high expression, respectively. Two pathologists evaluated and scored IHC results blindly.

### 2.3. Statistical analysis

The correlation between CXCL17 and EMT markers and clinicopathological factors was evaluated by the χ^2^ test. Association between CXCL17 and EMT markers in cervical cancer was assessed using Spearman’s rank correlation test. Data are presented as the mean ± SD. Comparisons between different groups were analyzed using the Student’s two-tailed *t* test. *P* value of less than .05 was considered statistical significance. Statistical analysis was done with SPSS17.0 software (Chicago).

## 3. Results

### 3.1. CXCL17 and GPR35 are overexpressed in cervical cancer tissues

**Figure 1.**
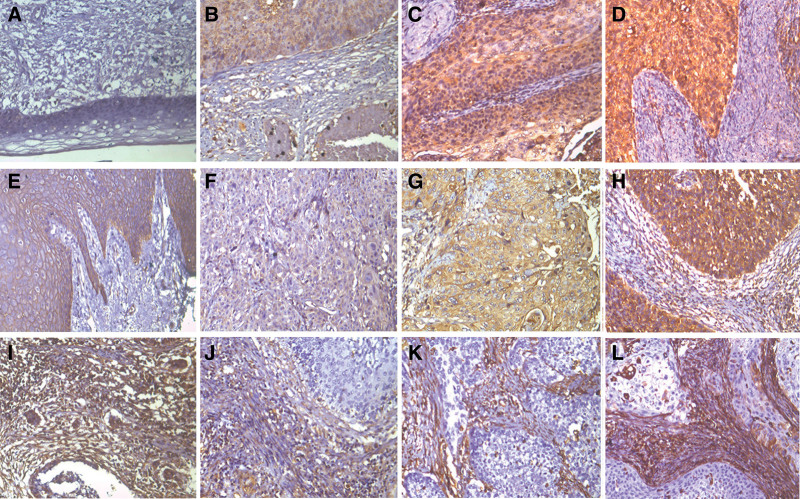
Expressions of CXCL17, E-cadherin, and vimentin in cervical cancer and normal cervical tissues. (A) No CXCL17 staining in normal cervical tissues apart from inflammatory cells. (B–D) Positive CXCL17 expression in cervical cancer tissues. (E) E-cadherin staining in normal cervical tissues. (F–H) Positive E-cadherin expression in cervical cancer tissues. (I) Vimentin staining was shown in stromal cells of normal cervical tissues. (J–L) Vimentin staining was also shown in some cancer cells near from stromal cells in cervical cancer tissues.

Firstly, we investigated the expression pattern of CXCL17 in 80 paraffinembedded cervical cancer tissues and 25 noncancerous cervical epithelial tissues by immunohistochemistry. As shown in Figure 1, CXCL17 was stained strongly in the infiltration of inflammatory cells and fibroblasts in the surrounding stroma of tumor. Notably, CXCL17 immunoreactivity was detected in the cytoplasm of cervical cancer cells (Fig. 1). In all 80 cervical cancer cases, CXCL17 expression was scored positively in 54 cases (67.50%), and 38 of them showed strong immunoreactivity. However, only 2 noncancerous cervical epithelial tissues had CXCL17 immunoreactivity in all 25 cases. Hence, there was a significant difference of CXCL17 expression between cervical cancer tissues and noncancerous cervical epithelial tissues.

**Figure 2.**
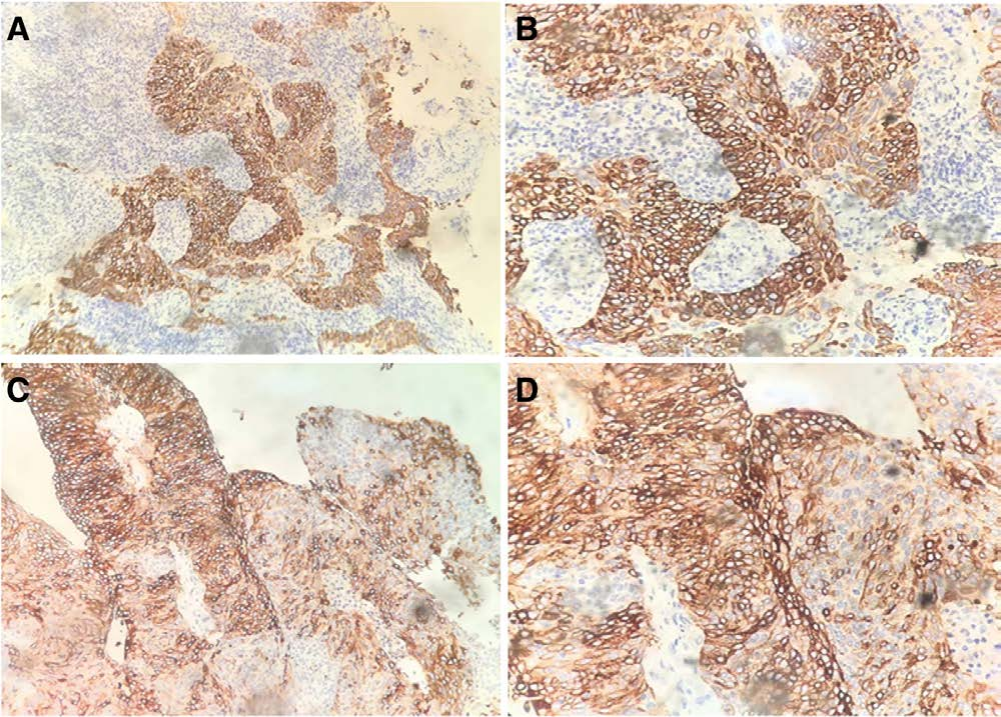
Immunohistochemical staining of GPR35 in cervical cancer samples. (A and C) Positive GPR35 expression in cervical cancer tissues (200×). (B and D) Positive GPR35 expression in cervical cancer tissues (400×).

As a receptor for CXCL17, we also detected the GPR35 expression pattern in cervical cancer tissues. As shown in Figure 2, GPR35 immunoreactivity was detected in the cytoplasm of cervical cancer cells. GPR35 expression was positive in 61 cases (76.25%) and negative in 19 cases (23.75%). Four noncancerous cervical epithelial tissues had GPR35 immunoreactivity in all 25 cases. Therefore, GPR35 expression was significantly enhanced in cervical cancer tissues.

### 3.2. CXCL17 overexpression is associates with lymph node metastasis

To clarify the clinical significance of CXCL17 expression in cervical cancer tissues, we study the association between CXCL17 expression and clinical parameters. Our results revealed CXCL17 expression was significantly correlated with lymph node metastasis and clinical stage (*P* < .05). In 38 cervical cancer samples with lymph node metastasis, 30 samples showed CXCL17 immunoreactivity, indicating CXCL17 might involve in metastasis of cervical cancer. However, there was no significant difference between CXCL17 expression and the other clinicopathological parameters, such as age and tumor size (*P* > .05).

### 3.3. Expression of E-cadherin, vimentin, and snail in cervical cancer tissues

**Figure 3.**
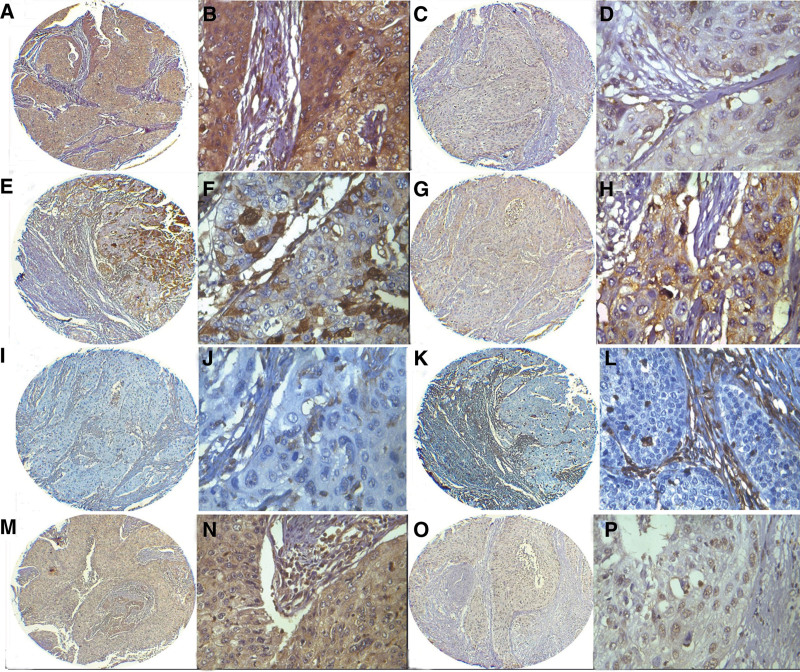
Immunohistochemical staining of CXCL17, E-cadherin, vimentin, and Snail in the same cervical cancer case. Strong positive expression of CXCL17 in cervical cancer (A and B). However, E-cadherin staining was weak in the same case (E and F). Vimentin expression was expressed in some cancer cells (I and J), and snail expression was enhanced in the same case (M and N). Accordingly, in CXCL17 weak case (C and D), E-cadherin staining was increased (G and H). Moreover, expressions of vimentin (K and L), and F-and snail (O and P) were all decreased in the same case.

To explore the expression patterns of EMT markers in cervical cancer, we examined a series of classical proteins, including E-cadherin, vimentin, and snail. Generally, E-cadherin protein was expressed only in the membranes of cervical squamous epithelium, whereas vimentin immunoreactivity was located in cytoplasm of stromal cells (Fig. 3). Compared with their normal counterpart, expression level of E-cadherin was obviously decreased in cervical cancer tissues. In all 80 cervical cancer cases, 58 cases showed positive staining of E-cadherin, and 22 of them showed thorough loss of E-cadherin immunoreactivity. In 58 positive staining cases, 42 displayed low expression of E-cadherin. Vimentin staining was mainly observed in the cytoplasm of stromal cells. It is noted that vimentin expression also displays scattered staining pattern on the edge of cancer cells in 46.25% cases (37/80). Moreover, 23 cases showed high expression of vimentin in scattered cancer cells. Snail, the vital transcription factor of inducing EMT, is mainly expressed in nuclear of cervical cancer cells. In all 80 cervical cancer cases, 52.50% (42) cases displayed positive snail immunoreactivity and 33 cases showed high expression of snail.

### 3.4. Correlation of CXCL17 expression with EMT markers

To elucidate the correlation between CXCL17 expression and EMT in cervical cancer, we performed immunostaining patterns of CXCL17 and relevant EMT markers, including E-cadherin, vimentin, and snail. In this assay, only 34 cases had E-cadherin immunoexpression in 54 cases with CXCL17 positive expression.

Moreover, 2 cases had no CXCL17 immunoreactivity in 22 cases with E-cadherin negative expression. Statistics revealed a significant negative correlation between CXCL17 and E-cadherin expression (*r* = −2.857, *P* = .005). In contrast, both CXCL17 and vimentin had immunoexpression in 33 cases. Whereas, neither CXCL17 nor vimentin was expressed in 22 cases. Therefore, CXCL17 had a positive correlation with vimentin expression (*r* = 0.430, *P* = .000). Simultaneously, as shown in Table 2, CXCL17 was significantly correlated with snail expression (*r* = 0.302, *P* = .006). These results demonstrated the close correlation between CXCL17 expression and EMT markers.

**Table 2:** Correlation of CXCL17 expression and EMT markers in cervical cancer tissues.

Variables	N	CXCL17 expression		*P* value
		Positive	Negative	
E-cadherin				
Positive	58	34	24	
Negative	22	20	2	.005*
Vimentin				
Positive	37	33	4	
Negative	43	21	22	.000*
Snail				
Positive	42	34	8	
Negative	38	20	18	.006*

* <.05.

## 4. Discussion

There exist a number of publications describing the correlation between the CXCL17 expression and the progression of various types of cancer.^[7–12]^ For example, Koni et al^[15]^ reported that CXCL17 overexpression increases migration and invasion of lung adenocarcinoma cells. This is the first study, to the best of our knowledge, investigates the correlation between CXCL17 expression and cervical cancer tissues. Our results showed that CXCL17 expression was mainly expressed in cytoplasm of tumor cells apart from detecting in leukocytes of tumor stroma. Our experiments showed that CXCL17 expression was significantly correlated with lymph node metastasis and clinical stage, highlighting the vital role of CXCL17 in invasion and metastasis of cervical cancer. Moreover, we also found GPR35 expression was enhanced in cervical cancer tissues in the present study. Our results indicate that CXCL17/GPR35 axis maybe participate in occurrence and development of cervical cancer.

EMT is known as a critical mechanism of epithelial cells acquire malignant mesenchymal phenotypes, thereby allowing tumor cells to migrate from the primary tumor.^[16,17]^ A series of factors have the potential ability to trigger EMT, including some inflammation-related cytokines derived from tumor cells and inflammatory cells. The inflammation-related cytokines could directly augment activity of transcriptional repressors of E-cadherin. Then, we also examined CXCL17 protein expression and EMT-related markers in cervical cancer samples by immunohistochemical staining. The most important characteristic of EMT is the loss of E-cadherin, which mediate epithelial junctions between adjacent epithelial cells.^[17]^ Down-regulation of E-cadherin leads to loss of epithelial morphology and transforms epithelial cells to more invasive mesenchymal cells. Vimentin, the symbol of mesenchymal marker for EMT, has been detected a variety of cancers.^[18,19]^ Snail1, a member of snail family of zinc-finger transcription factors, is also a key regulator of EMT.^[20]^ In this study, we revealed a significant negative correlation between CXCL17 and E-cadherin expression. In contrast, CXCL17 had a positive correlation with vimentin expression. Furthermore, CXCL17 was significantly correlated with snail expression. These results demonstrated the close correlation between CXCL17 expression and EMT markers. Taking into account the role of CXCL17 in lymph node metastasis and higher clinical stage, CXCL17 might be contribute to progression and metastasis of cervical cancer by EMT.

In conclusion, CXCL17 expression could be a latent marker of tumor progression and EMT in cervical cancer. CXCL17 may contribute to invasion and metastasis of cervical cancer via EMT. Moreover, CXCL17/GPR35 axis maybe participate in occurrence and development of cervical cancer. Further investigation of the molecular mechanisms underlying the role of CXCL17 in the process of EMT in cervical cancer is needed. Our results provides a new viewpoint about progression of cervical cancer and an effective therapeutic target for treatment of this disease.

## Author contributions

**Conceptualization:** Jianguang Wang.

**Data curation:** Tianshu Pang, Xiaokang Zhang, Longhui Zhang.

**Investigation:** Tianshu Pang, Xiaokang Zhang.

**Project administration:** Jingming Zhai, Like Zhang, Lujia Dong.

**Resources:** Jingming Zhai.

**Validation:** Jingming Zhai, Tianshu Pang, Yanhui An.

**Visualization:** Yanhui An.

**Writing – original draft:** Jingming Zhai, Tianshu Pang, Yanhui An.

**Writing – review & editing:** Jingming Zhai.
